# Association of extent of resection on recurrence-free survival and functional outcome in vestibular schwannoma of the elderly

**DOI:** 10.3389/fonc.2023.1153698

**Published:** 2023-06-05

**Authors:** Sophie Shih-Yüng Wang, Kathrin Machetanz, Florian Ebner, Georgios Naros, Marcos Tatagiba

**Affiliations:** ^1^ Department of Neurosurgery and Neurotechnology, Eberhard Karls University Tübingen, Tübingen, Germany; ^2^ Department of Neurosurgery, Alfried Krupp Hospital, Essen, Germany

**Keywords:** acoustic neuroma, neuro-oncology, skull base, geriatric, elderly, vestibular schwannoma

## Abstract

**Background:**

Despite the ongoing debate on the risk–benefit ratio of vestibular schwannoma (VS) treatment options, watchful observation and radiation are usually favored in the elderly (>65 years). If surgery is inevitable, a multimodal approach after deliberate subtotal resection has been described as a valid option. The relationship between the extent of resection (EOR) of surgical and functional outcomes and recurrence-free survival (RFS) remains unclear. This present study aims to evaluate the functional outcome and RFS of the elderly in relation to the EOR.

**Methods:**

This matched cohort study analyzed all consecutive elderly VS patients treated at a tertiary referral center since 2005. A separate cohort (<65 years) served as a matched control group (young). Clinical status was assessed by the Charlson Comorbidity Index (CCI), the Karnofsky Performance (KPS), and the Gardner and Robertson (GR) and House & Brackmann (H&B) scales. RFS was evaluated by Kaplan–Meier analysis using contrast-enhanced magnetic resonance imaging to identify tumor recurrence.

**Results:**

Among 2,191 patients, 296 (14%) patients were classified as elderly, of whom 133 (41%) were treated surgically. The elderly were characterized by a higher preoperative morbidity and worse gait uncertainty. Postoperative mortality (0.8% and 1%), morbidity (13% and 14%), and the functional outcome (G&R, H&B, and KPS) did not differ between the elderly and the young. There was a significant benefit in regard to the preoperative imbalance. Gross total resection (GTR) was accomplished in 74% of all cases. Lower grades of the EOR (subtotal and decompressive surgery) raised the incidence of recurrence significantly. Mean time to recurrence in the *surg*ELDERLY was 67.33 ± 42.02 months and 63.2 ± 70.98 months in the *surgCONTROL*.

**Conclusions:**

Surgical VS treatment aiming for complete tumor resection is feasible and safe, even in advanced age. A higher EOR is not associated with cranial nerve deterioration in the elderly compared to the young. In contrast, the EOR determines RFS and the incidence of recurrence/progression in both study cohorts. If surgery is indicated in the elderly, GTR can be intended safely, and if only subtotal resection is achieved, further adjuvant therapy, e.g., radiotherapy, should be discussed in the elderly, as the incidence of recurrence is not significantly lower compared to the young.

## Introduction

Among benign nerve sheath tumors, the vast majority are vestibular schwannomas (VSs) with a reported incidence rate of 1.52 per 100,000 (1, 2). They account for 80%–90% of all tumors in the cerebellopontine angle and approximately 6%–8% of all primary intracranial neoplasms (3, 4). Historically, surgical removal has been an appreciated treatment of choice as complete resection represents maximal tumor control (5). However, due to the anatomical relationship of the VS to multiple cranial nerves (CNs), a great deal of precision and delicacy is required in surgical management (1, 6). Several studies have documented severe clinical consequences of postoperative CN function decline for VS patients (1, 7). Thus, other treatment options including watchful observation (i.e., wait-and-scan, WaS) and radiation (i.e., stereotactic radiosurgery, SRS) have been claimed (8). Ever since, there is an ongoing debate on the risk–benefit ratio of these treatment options on tumor control and function preservation considering different factors such as tumor size, initial CN function, and/or patient’s age (9).

In contrast to other patient cohorts, there seems to be a general agreement that watchful observation and radiation treatment should be favored in the elderly (>65 years of age) assuming a higher operative morbidity and lower life expectancy (10, 11). However, surgical intervention is sometimes inevitable, even in this patient cohort (e.g., large VS compressing the brainstem). In these particular cases, a multimodal therapy approach [i.e., deliberate subtotal resection (STR) with adjuvant SRS] has been suggested as a valid option recently in order to reduce perioperative morbidity (9). The level of evidence, however, is remarkably low (9).

However, creating clinical evidence in VS management, the *elderlies* (>65 years of age) is of paramount importance, as an incidence peak is described in this specific patient group (9). In detail, there are three evidence gaps for the elderly patient cohort. Firstly, the few existing small-sized studies on surgical morbidity in the elderly describe the outcome of surgeries performed several decades ago (10–12) and surgical techniques have evolved reducing surgery time and improving functional outcome since then (8, 13). Thus, it remains unclear whether the surgical morbidity of the elderly differs from the young in the environment of contemporary neurosurgery. Second, as deliberate STR or decompressive surgery (DS) could be a valid option in this specific subgroup, the relation of surgical morbidity to the extent of resections (EORs) in the elderly should be investigated. Third, there is no data on the relation between the EOR on tumor control as in long-term recurrence-free-survival (RFS) (i.e. progress-free-survival in subtotal resection) in the elderly. Notably, most studies provide follow-up data of <5 years (10, 11).

Thus, the aim of this study is to evaluate (1) the patterns of VS management in a tertiary neurosurgical center, (2) the onco-functional outcome, and (3) the incidence of recurrence in relation to the EOR in a large cohort of elderly patients compared to a matched-control cohort.

## Methods

### Study design and patient cohort

**Figure 1:**
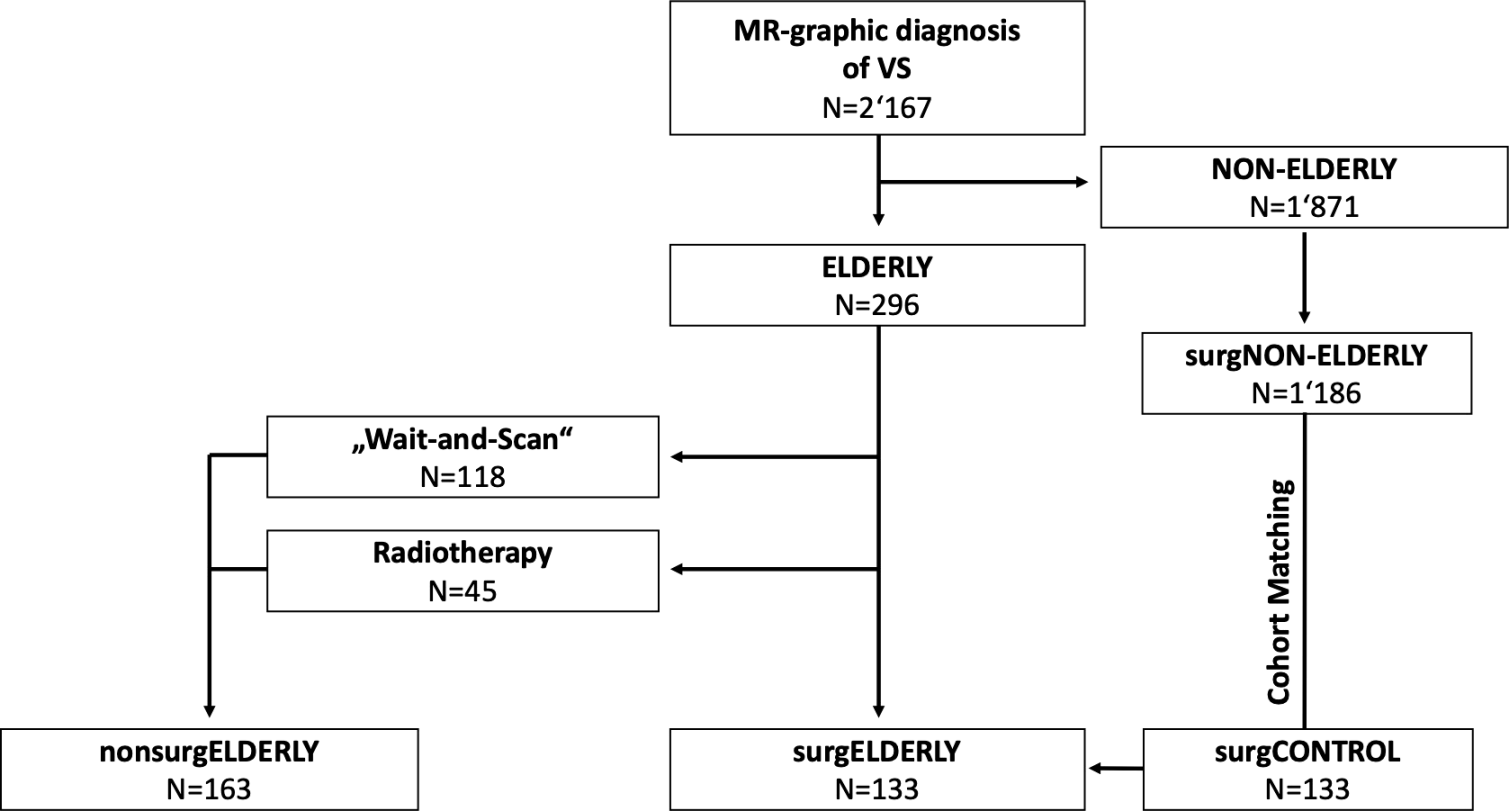
Flow chart on the study population.

This retrospective blinded cohort study analyzed all consecutive (elderly) patients (>65 years) with unilateral VS treated at a German academic, tertiary referral center between April 2005 and October 2020. Patients were referred to non-surgical (i.e., WaS, stereotactic radiosurgery; *nonsurg*ELDERLY) or surgical treatment (*surg*ELDERLY) depending on tumor size, the presence of hydrocephalus, clinical presentation, and a patient’s individual preference. A separate cohort of patients with <65 years of age, who underwent elective VS surgery, served as a matched control group (*surgCONTROL*) to specifically compare surgical treatment in the elderly and the young (**Figure 1**). Pairing was based on the *EOR*, surgical approach (semi-sitting or prone position), gender, and tumor size (closest match). All patients were treated by the retrosigmoid approach with intraoperative neurophysiological monitoring. Large VS (e.g., Koos °III–°IV) were generally treated in the semi-sitting position, while small VS (e.g., Koos °I–°II) in the prone position (14). VS associated with neurofibromatosis was systematically excluded from this study. Same was applied to the previously treated VS (by surgery or radiotherapy). All histopathological examinations of *surg*ELDERLY and *surgCONTROL* were graded as schwannoma by a board-certified neuropathologist. The study population was identified through a prospective registry. The local ethics committee approved data collection and *post-hoc* analyses.

### Data collection

**Figure 2:**
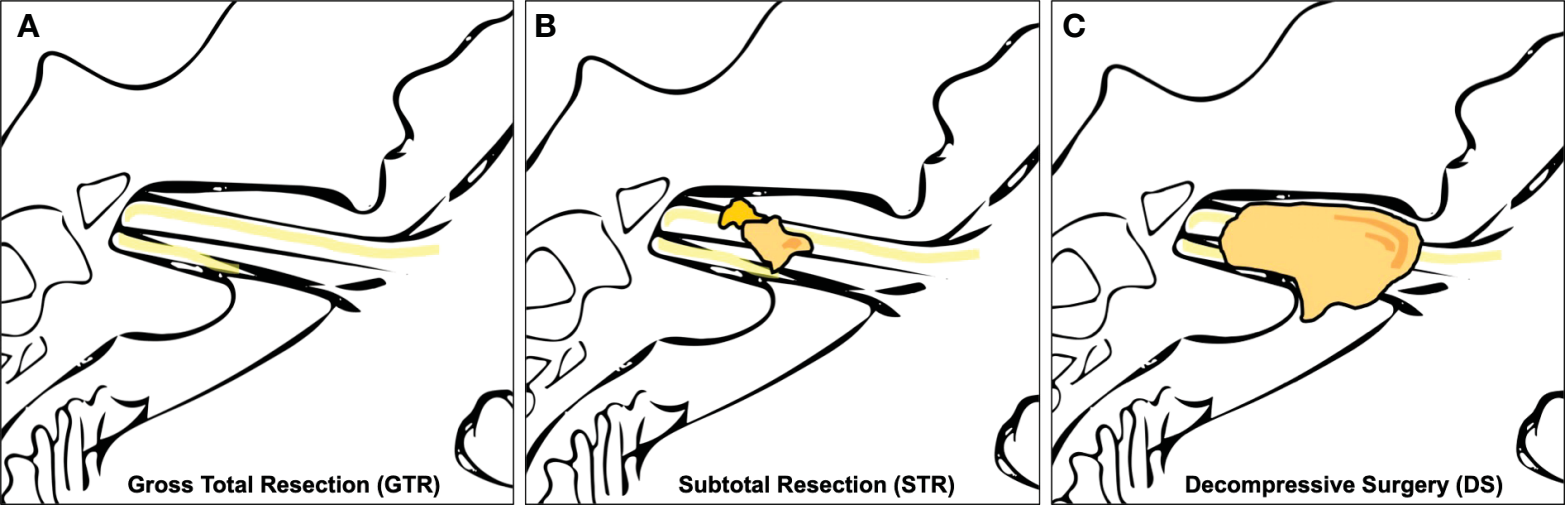
Extent of resection (EOR). **(A)** shows gross total resection (GTR), **(B)** subtotal resection (STR), and **(C)** shows a schematic representation of decompressive surgery (DS) with no decompression of the tumor in the internal auditory canal.

Medical records of each patient were reviewed, and various demographic, tumor, and treatment variables were recorded. Magnetic resonance images (MRIs) were retrospectively analyzed to determine the tumor size according to the Koos grading system in a blinded fashion (14). The EOR was determined by postoperative MRI (3 months postoperatively) and classified by gross total resection (GTR), STR (i.e., residual tumor exclusively in the internal auditory canal), and DS (i.e., residual tumor beyond the internal auditory canal) (**Figure 2**). MR-graphic tumor progression/recurrence was defined as tumor progress or new tumor recurrence during MR-graphic surveillance with gadolinium contrast. Symptom-affected everyday-life dependency was acquired with the Karnofsky Performance Score (KPS) (15). Pre- and postoperative symptoms were recorded including tinnitus, functional hearing loss in Gardner–Robertson (GR) classes (16), gait uncertainty, vertigo, trigeminal affection (neuralgia or hypesthesia), double vision, swallowing deficit, headache, gustatory deficit, hydrocephalus, and facial palsy. Facial nerve function was reported using the House and Brackmann (H&B) scale pre- and postoperatively, as well as after a follow-up of 3 months and 1 year (17). Recorded patient comorbidities were assessed using the Charlson Comorbidity Index (CCI) (18). Adverse postoperative events were classified according to the Clavien–Dindo Classification (CDC) (19).

### Statistical analysis

Statistical analysis was performed in R Studio (Version 1.2) using descriptive statistics. To compare nonnumeric parameters of both groups, the chi-square test and Fisher’s exact test were applied. For numeric parameters, Welch’s two-sample *t*-test was used. Recurrence-free and overall survival were estimated using the Kaplan–Meier method and compared between cases and controls using a log-rank test. The length of follow-up for recurrence-free survival was calculated from the date of surgical intervention to the date of either recurrence or the last clinical visit. Significance was defined as the probability of a two-sided type 1 error being <5% (p < 0.05). Data are presented as mean ± standard deviation (SD) if not indicated otherwise. Due to the low incidence of complications and perioperative morbidity, for its analysis and comparison dependent on the EOR, DS and STR were grouped together.

## Results

### Study cohorts

Among 2,167 patients with VS, 296 patients (14%) were of >65 years of age at date of diagnosis and classified as elderly (**Figure 1**). Mean age was 71.1 ± 5.0 [range 65–89] years in all elderly patients. Tumor size was equally distributed across Koos grades (°I: 69/296, 23%; °II: 86/296, 29%; °III: 80/296, 27%; °IV: 61/296. 21%). The majority of elderly patients (163/296, 55%) was managed non-surgically (*nonsurg*ELDERLY) by either SRS (45/163, 28%) or watchful observation (118/163, 72%). 133/296 (45%) of elderly patients were treated microsurgically (*surg*ELDERLY) *via* a retrosigmoid approach. A separate cohort of patients younger than 65 years (N = 133) served as a matched control group (*surgCONTROL*). The mean age of the *surgCONTROL* cohort was 46.3 ± 11.9 years (**Table 1**).

**Table 1:** Patient demographics, tumor characteristics, and initial clinical presentation.

	nonsurgELDERLY	surgELDERLY	surgCONTROL	p-value**
***No. of cases***	163	133	133	N/A
Demographics				
*Age*	71.9 ± 5.1	70.2 ± 4.6	46.3 ± 11.9	0.002/<0.001*
*Female*	95 (58)	72 (54)	61 (46)	0.717/0.220
Tumor size				
*Koos°I*	67 (41)	2 (2)	2 (2)	<0.001*/1
*Koos°II*	64 (39)	22 (16)	22 (16)	<0.001*/1
*Koos°III*	27 (17)	53 (40)	53 (40)	<0.001*/1
*Koos°IV*	5 (3)	56 (42)	56 (42)	<0.001*/1
***MR-graphic tumor progression***	30 (18)	31 (23)	25 (19)	0.371/0.081
***Cystic tumor***	5 (3)	12 (9)	8 (6)	0.052/0.486
***KPS score***	82.9 ± 10.2	81.1 ± 8.4	85.4 ± 6.5	0.105/<0.001*
Initial neurological symptoms				
*Facial palsy*	6 (4)	21 (16)	10 (8)	<0.001*/0.056
*Tinnitus*	45 (28)	39 (29)	59 (44)	0.840/0.016*
*Functional hearing loss*	71 (53)	93 (70)	101 (76)	<0.001*/0.334
*Gait uncertainty*	33 (20)	52 (39)	18 (14)	<0.001*/<0.001*
*Vertigo*	62 (38)	55 (41)	40 (30)	0.644/0.073
*Trigeminal affection*	4 (2)	20 (15)	22 (17)	<0.001*/0.867
*Swallowing deficit*	0 (0)	5 (4)	3 (2)	0.041*/0.719
*Headache*	0 (0)	1 (1)	0 (0)	N/A/0.316
*Other*	0 (0)	0 (0)	2 (2)	N/A/0.478
Comorbidities				
*Charlson Index*	0.45 ± 0.90	0.6 ± 1.11	0.1 ± 0.44	0.176/<0.001*
*0*	123 (75)	94 (70)	126 (94)	0.360/<0.001*
*1*	18 (11)	13 (10)	2 (2)	0.870/0.045*
*2*	12 (7)	16 (12)	4 (3)	0.243/0.011*
*>3*	10 (7)	10 (8)	1 (1)	0.646/0.014*

(**) p-values indicate significant differences comparing (i) nonsurgELDERLY vs. surgELDERLY and (ii) surgELDERY vs. surgCONTROL cohorts. Values are presented as the number of patients (%) unless indicated otherwise. Significant p-values (<0.05) are highlighted in bold. p-values are indicated as *nonsurgELDERLY* vs. *surgELDERLY/surgELDERLY* vs. *surgCONTROL*.

N/A = Not applicable. "*" signifies a statistical significant value.

The most common initial symptom in the elderly was functional hearing loss in 172/296 (58%) cases, followed by vertigo in 117/296 (40%) patients. Tinnitus and gait uncertainty were similarly common with 84/296 (28%) cases and 85/296 (29%) cases, respectively. Facial palsy was a leading initial symptom in only 27/296 (9%) cases in the elderly. Trigeminal affection and swallowing deficits were very rare clinical symptoms with 24/296 (8%) and 5/296 (2%) cases, respectively. No patient presented with double vision (**Table 1**).

When comparing *surg*ELDERLY with the younger *surgCONTROL* cohort, they presented with a worse initial KPS (81.1 ± 8.4 and 85.4 ± 6.5, respectively; p < 0.001) poorer preoperative comorbidity status (e.g., the incidence of myocardial infarction, congestive heart failure, diabetes, and malignant tumors), yielding in a significantly higher CCI in the *surg*ELDERLY compared to the *surgCONTROL* group (0.6 ± 1.1 and 0.1 ± 0.4, respectively; p < 0.001) (**Tables 1**, **2**). However, there was no significant difference in the following VS-associated morbidities: initial incidence of facial palsy, functional hearing loss, vertigo, trigeminal affection, swallowing deficit, headache, gustatory deficit, and hydrocephalus. *surg*ELDERLY had a higher incidence of pre-operative gait uncertainty (p < 0.001), but complained of less tinnitus than their controlled matches (p = 0.016).

**Table 2:** Patients’ comorbidities (*surgELDERLY vs. surgCONTROL*).

Comorbidities	surgELDERLY	surgCONTROL	p-value
*Myocardial infarction*	9 (7)	0 (0)	**0.007**
*Congestive heart failure*	6 (5)	0 (0)	**0.039**
*Peripheral vascular disease*	1 (1)	0 (0)	0.316
*Cerebrovascular disease*	8 (6)	3 (2)	0.218
*Dementia*	1 (1)	0 (0)	0.316
*Peptic ulcer disease*	1 (1)	0 (0)	0.316
*Mild liver disease*	2 (2)	0 (0)	0.478
*Diabetes, uncomplicated*	6 (5)	0 (0)	**0.039**
*Hemiplegia*	1 (1)	1 (1)	1
*Renal disease*	2 (2)	0 (0)	0.478
*Tumor without metastasis*	15 (11)	4 (3)	**0.017**
*Metastatic tumor*	3 (2)	0 (0)	0.245

Values are presented as the number of patients (%) unless indicated otherwise. Significant p-values (<0.05) are highlighted in bold.

### Non-surgical vestibular schwannoma management in the elderly

The *surg*ELDERLY and *nonsurg*ELDERLY cohort did not differ in age, gender, the clinical status in KPS. The incidence of cystic morphology and MR-graphic progression was higher in the surgically treated but did not reach statistical significance in this cohort. However, non*surg*ELDERLY were characterized by a significant smaller tumor size (p < 0.001). The non*surg*ELDERLY cohort had less initial severe CN deficits (facial palsy, functional hearing loss, gait uncertainty, trigeminal affection) than *surg*ELDERLY. The CCI was not significantly different in both cohorts (**Table 1**).

### Surgical vestibular schwannoma management in the elderly

The *surgCONTROL* was younger than the *surgELDERLY* cohort but did not differ in other patient demographics or tumor characteristics (**Table 1**). *surg*ELDERLY presented with a worse initial KPS then their younger *surgCONTROL* cohort (81.1 ± 8.4 and 85.4 ± 6.5, respectively; p < 0.001). There were no significant group difference in the initial incidence of facial palsy, functional hearing loss, vertigo, trigeminal affection, swallowing deficit, headache, gustatory deficit and hydrocephalus in a matched control comparison (“*surg*ELDERLY” vs. “*surgCONTROL*”). Remarkably, *surgELDERLY* had a higher incidence of gait uncertainty but complained of less tinnitus than their controlled matches. Additionally, *surg*ELDERLY had a poorer preoperative comorbidity status (e.g., the incidence of myocardial infarction, congestive heart failure, diabetes, and malignant tumors), yielding in a significantly higher CCI in the *surg*ELDERLY compared to the *surgCONTROL* group (0.6 ± 1.1 and 0.1 ± 0.4, respectively; p < 0.001) (**Tables 1**, **2**).

### Surgical data and surgical complications

Surgery was performed by a retrosigmoid craniotomy in all cases using either a semi-sitting (221/266, 83%) or prone position (45/266, 17%) in both surgical study groups. Mean operating time (skin to skin) was noted similarly at 248.0 ± 75.2 minutes in the *surg*ELDERLY and 240.0 ± 82.1 minutes in the *surgCONTROL* (p = 0.398).

The incidence of perioperative complication was comparable in the *surg*ELDERLY (17/133, 13%) and *surgCONTROL group (19/133, 14%)* (p=0.858). Larger tumors (Koos °III and °IV) more often yielded in postoperative complications (*surg*ELDERLY: °II: N = 3; °III: N = 2; °IV: N = 12 and *surgCONTROL*: °I: N = 1; °II: N = 2; °III: N = 8; °IV: N = 8). Postoperative hemorrhage, venous thrombosis and symptomatic pneumoncephalon occurred more frequently in the *surg*ELDERLY, but this did not reach any statistical significance. In contrast, younger patients were more prone to postoperative CSF leakage. Overall, the complication rate in the *surg*ELDERLY cohort was not significantly raised. One patient suffered a postoperative hemorrhage and treatment was terminated after the patient’s presumed will, yielding in a mortality rate of N = 1 (**Table 3**). The CDC Index of complication severity is summarized in **Table 4**. Discharge modality (home, rehab, and other hospital) was significantly indifferent in both groups.

**Table 3:** Incidence of perioperative complication surgELDERLY and surgCONTROL.

Incidence of complications following VS surgery	surgELDERLY	surgCONTROL	p-value
*Mortality*	1 (0.8)	0 (0)	0.316
*Postoperative neurological complications (including secondary to infarction or hemorrhage)*	4 (3)	0 (0)	0.131
*Hydrocephalus*	4 (3)	1 (1)	0.366
*CSF otorrhea/rhinorrhea*	7 (5)	15 (11)	0.119
*Ventriculostomy placement*	2 (1)	1 (1)	0.562
*Facial nerve reconstruction*	4 (3)	1 (1)	0.366
*Symptomatic pneumencephalon*	3 (2)	0 (0)	0.245
*Sinus thrombosis*	1 (1)	3 (2)	0.614

Values are presented as the number of patients (%) unless indicated otherwise.

**Table 4:** Complication severity according to the Clavien–Dindo Classification.

Clavien–Dindo Classification	surgELDERLY	surgCONTROL	p-value
***0***	116 (87)	114 (86)	0.685
***1***	1 (1)	1 (1)	1
***2***	1 (1)	3 (2)	0.290
***3***	14 (11)	14 (11)	1
*3a*	5 (36)	10 (71)	0.065
*3b*	9 (64)	4 (29)	
***4***	0 (0)	1 (1)	0.158
*4a*	0 (0)	1 (100)	0.158
*4b*	0 (0)	0 (0)	
***5***	1 (1)	0 (0)	0.301
*Total incidence*	17 (13)	19 (14)	0.858

Values are presented as the number of patients (%) unless indicated otherwise. Significant p-values (<0.05) are highlighted in bold.

### Functional outcome

Postoperative KPS did not differ between *surg*ELDERLY and *surgCONTROL* patients (78.1 ± 9.3 and 78.8 ± 4.9; p = 0.409). There was no significant difference in discharge modality (p = 0.377). Impeccable facial function (H&B = 1) was completely unaffected by the surgery in 38/112 (34%) and 53/123 (43%) in *surg*ELDERLY and *surgCONTROL*, respectively (p = 0.096). After surgery-related deterioration, facial function recovered to a favorable outcome (i.e., H&B°I and H&B°II) in 45/75 (60%) and 46/70 (65%) *surg*ELDERLY and *surgCONTROL* patients, respectively (**Table 5**). Therefore, overall favorable facial function outcome after 1 year was 70% (93/133) in the elderly and 76% (102/133) in the young. Hearing preservation was insignificantly better in *surg*ELDERLY at 84% (27 out of 32 with functional hearing) than in *surgCONTROL* at 71% (28 out of 39 with functional hearing) with p = 0.164. New trigeminal deficit was observed in 2%. Of the 20 patients suffering from preoperative trigeminal affection (hypesthesia or neuralgia), 17 patients recovered clinically in the *surg*ELDERLY group (85%); the same was observed in 20/22 patients (90%) in the *surgCONTROL* group (p = 0.277). New postoperative gait uncertainty was observed in N = 4 in the *surg*ELDERLY but none in the younger *surgCONTROL* group. Postoperative recovery of symptomatic gait uncertainty was observed in 71% (37/52) and 76% (12/16) of the *surg*ELDERLY and *surgCONTROL*, respectively (p = 0.382). The recovery of vertigo symptoms was observed in N = 40/55 (71%) of all *surg*ELDERLY presenting with preoperative vertigo; this rate was higher in the *surgCONTROL* cohort with N = 37/40 (90%) (p = 0.013). The incidence of new postoperative vertigo was N = 0 (0%) in *surg*ELDERLY but N = 8/91 (9%) in its control cohort. Considering new postoperative tinnitus, the incidence was higher in the young (N = 1 in *surgELDERLY* and N = 9 in *surgCONTROL)*, whereas the postoperative improvement of known tinnitus was similar at 14% and 16%, respectively (*surg*ELDERLY and *surgCONTROL*) (p = 0.585).

**Table 5:** Functional outcome.

	surgELDERLY	surgCONTROL	p-value
***KPS at discharge***	78.05 ( ± 9.25)	78.79 ( ± 4.93)	0.409
***Shunt dependency***	8 (6)	2 (2)	0.104
***Tumor recurrence***	5 (4)	10 (8)	0.288
***Initial House–Brackmann***	1.28 ( ± 0.74)	1.14 ( ± 0.55)	0.075
*H&B I*	112 (84)	123 (92)	
*H&B II*	10 (8)	5 (4)	
*H&B III*	8 (6)	3 (2)	
*H&B IV*	1 (1)	1 (1)	
*H&B V*	2 (1)	1 (1)	
*H&B VI*	0 (0)	0 (0)	
***Postoperative House–Brackmann***	2.64 ( ± 1.37)	2.39 ( ± 1.44)	0.150
*H&B I*	38 (29)	53 (40)	
*H&B II*	27 (20)	28 (21)	
*H&B III*	26 (20)	13 (10)	
*H&B IV*	31 (23)	26 (19)	
*H&B V*	9 (7)	12 (9)	
*H&B VI*	2 (1)	1 (1)	
***House–Brackmann 1 Year follow-up***	1.98 ( ± 1.29)	1.71 ( ± 1.11)	0.061
*H&B I*	73 (55)	88 (66)	
*H&B II*	20 (15)	14 (10)	
*H&B III*	16 (12)	19 (14)	
*H&B IV*	18 (13)	10 (8)	
*H&B V*	5 (4)	2 (2)	
*H&B VI*	1 (1)	0 (0)	
Initial Gardner–Robertson Grade			
*I–II (serviceable)*	32 (24)	39 (29)	0.209
*III–IV (non-serviceable)*	101 (76)	94 (71)	
Postoperative Gardner–Robertson Grade			
*I–II (serviceable)*	27 (20)	28 (21)	0.843
*III–IV (non-serviceable)*	106 (80)	105 (79)	
Discharge to			
*Home*	126 (95)	131 (98)	0.376
*Rehab*	3 (2)	1 (1)	
*Other hospital*	3 (2)	1 (1)	
*Death*	1 (1)	0 (0)	

Values are presented as the number of patients (%) unless indicated otherwise. Significant p-values (<0.05) are highlighted in bold.

### Extent of resection and recurrence-free survival

GTR was achieved in the majority of *surg*ELDERLY cases (99/133, 74%). When taking the EOR into account, there was no significantly higher postoperative CN affection (facial, trigeminal, and vestibulocochlear) in *surg*ELDERLY associated with GTR and STR/DS (**Table 6**). However, the rate of permanent facial deterioration within patients treated with GTR remained significantly higher in the *surg*ELDERLY compared to the *surgCONTROL* (p = 0.035). Additionally, within the *surgCONTROL* group, STR/DS was associated with a significantly higher incidence of permanent facial deterioration (p = 0.036) compared to GTR.

**Table 6:** Comparison between the extent of resection.

	GTR (N = 99) [surgELDERLY/surgCONTROL]	STR & DS (N = 34) [surgELDERLY/surgCONTROL]	p-value [surgELDERLY/surgCONTROL]
*Recurrence*	1 (1)/3 (3)	4 (12)/7 (21)	0.015*/0.003
*Complications*	13 (13)/14 (14)	4 (12)/4 (12)	1/1
*Hearing loss*	4 (4)/10 (10)	0 (0)/1 (3)	0.572/0.288
*New tinnitus*	1 (1)/8 (8)	0 (0)/1 (3)	1/0.447
*Temporary facial deterioration*	24 (24)/30 (30)	10 (29)/8 (24)	0.649/0.515
*Permanent facial deterioration*	33 (34)/19 (19)	8 (24)/13 (38)	0.389/**0.036**

Values are presented as the number of patients (%) unless indicated otherwise. Significant p-values (<0.05) are highlighted in bold.

**Figure 3:**
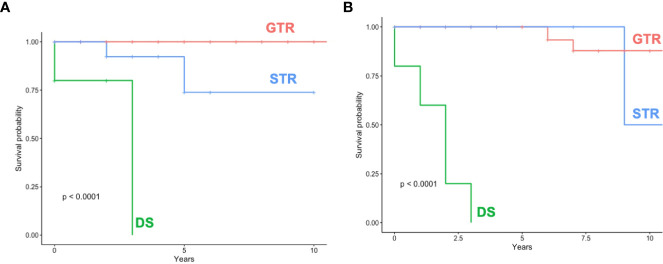
Recurrence/Progression-free-survival (RFS) **(A)** shows RFS dependent on the EOR in *surg*ELDERLY and **(B)** surg*CONTROL*. GTR, gross total resection; STR, subtotal resection; DS, decompressive surgery.

STR/DS was significantly associated with the incidence of recurrence compared to GTR in both subgroups (*surg*ELDERLY: p = 0.015; *surgCONTROL*: p = 0.003). The incidence of recurrence was statistically insignificant in *surg*ELDERLY compared to *surgCONTROL*, when treated with GTR (p = 0.621). Mean time for surveillance was 38 ± 36 months in *surgCONTROL* (median: 25 months) and 31 ± 37 months (median: 34 months) in *surg*ELDERLY. The overall incidence for recurrence was 5/133 (4%) and 10/133 (8%) after neurosurgical tumor resection in the *surg*ELDERLY and *surgCONTROL*, respectively, with no statistical significance (p = 0.143). Mean time to recurrence was 67.33 ± 42.02 months in *surg*ELDERLY and 63.2 ± 70.98 months in *surgCONTROL*. Kaplan–Meier analysis on RFS depending on the EOR is shown in **Figure 3**. The EOR was significantly associated with RFS in both *surg*ELDERLY and *surgCONTROL* cohorts (p < 0.001). Mean time to recurrence in *surg*ELDERLY was 67 ± 53 months and 79 ± 71 months in *surgCONTROL*.

## Discussion

The present study aimed to evaluate the management of VS in the elderly (>65 years) in a tertiary neurosurgical center and to assess the oncological and functional outcome of this particular cohort in comparison to matched young controls and in regard to the EOR. The elderly represented approx. 14% of all VS patients in our cohort. The majority was eligible for non-surgical treatment. Patients were allocated for surgical treatment due to large tumor size affecting the brainstem and worse preoperative clinical symptomatology (e.g., the presence of vertigo and imbalance). In comparison to a matched-control cohort, elderly selected for surgery were characterized by a worse preoperative clinical condition (KPS and CCI). Despite this, postoperative mortality, morbidity and functional outcome did not differ between both surgical groups. These data suggest that the retrosigmoid approach for VS resection is safe in the elderly >65 years of age. Patients significantly benefit in regard to preoperative imbalance. GTR was accomplishable in the majority of patients. Notably, the EOR determined RFS.

### Patterns of care and clinical characteristics

In the present population of surgically treated elderly (*surg*ELDERLY), preoperative CN deficits (facial function, hearing, gait, and trigeminal affection) were distinctively more severe than in the conservatively managed (*nonsurg*ELDERLY). Same was described in a nationwide registry study in Sweden with 58 elderly patients and by a series of surgically managed elderly patients by Samii et al. (12, 20). When comparing the nuances of clinical deficits, *surg*ELDERLY rarely presented with tinnitus but suffered from gait uncertainty more often than their matched and younger cohort. Interestingly, preoperative functional hearing was similar in both groups and not decreased in the elderly, even though presbyacusis is a common phenomenon in the general elderly population (21). This observation suggests that symptoms leading to the diagnosis of VS most likely are not hearing function but other vestibulocochlear (gait and vertigo), facial, or trigeminal symptoms.

### Microsurgical care

Microsurgical tumor resection of VS by a retrosigmoid craniotomy was safe and did not yield in a higher risk for postoperative CN deficits in the elderly in comparison to the matched *surgCONTROL* cohort. Even with a highly significant incidence of relevant comorbidity (i.e., higher CCI and worse KPS), perioperative complication rates and morbidity are statistically comparable. Perioperative complications in the elderly have been described by current literature from 20% to 57% (1, 3, 10). In contrast, the present population showed an incidence of perioperative complication of 13% and 14% in the elderly and the young, respectively. Due to a higher case load of elderly VS patients in the past 15 years (i.e., nine cases per year) in comparison to previous studies (approx. two to three per year) (3, 10), our department was able to acquire and develop a surgical routine and protocols for VS surgery in this patient cohort. The operative experience of the surgical team has been described as an important factor affecting onco-functional outcome in VS management, and therefore, treatment in high-volume centers has been recommended (9). The distribution of complications is different in both cohorts with a higher prevalence of CSF leakage and venous thrombosis in the younger population, while the elderly more often suffered from postoperative hemorrhage and symptomatic pneumocephalus, which has been described in the literature before (1, 10, 12, 22). Previous studies have mixed surgical approaches (translabyrinthine, middle fossa, and retrosigmoid) (23). This is the first comparative series to describe a large elderly cohort treated exclusively by a microsurgical, retrosigmoid approach. Thus, lower perioperative morbidity of the present study might be partially attributed by differences in the surgical approach.

### Postoperative functional outcome

There were no differences in general functional outcome or the independency score (KPS) comparing the *surg*ELDERLY to the *surgCONTROL*. In contrast, the *surg*ELDERLY cohort showed a significant improvement of gait/vertigo after surgery. Tinnitus was improved in 13%–14% of all surgically treated. Postoperative hearing loss was not worse but significantly better in the *surg*ELDERLY. In approx. 60% of the patients, facial function normalized within 3 months, making postoperative functional deterioration temporary. There was no difference in facial nerve outcome between the *surg*ELDERLY and the *surgCONTROL* group. While observing a remarkable transient functional deterioration right after surgery, of which the majority recovers, these numbers should not be compared to direct results after other non-surgical treatment options (e.g., radiotherapy or radiosurgery). The rate of permanent facial deterioration was significantly higher in *surg*ELDERLY compared to *surgCONTROL* in the GTR group suggesting decreased postoperative rehabilitation potential in facial function with advanced age (24).

The explanation for the inversely proportional relationship of permanent facial deterioration and GTR vs. STR/DS in the elderly (more facial deterioration in GTR) and the young (more facial deterioration in STR/DS) could be attributed to the actual intention-to-treat. In the young, we usually intend GTR unless there is a deterioration of the intraoperative neuromonitoring (e.g., facial motor–evoked potentials). This approach explains the GTR rate of 74% in the present study. However, in a larger cohort of the same group, GTR was even higher with approx. 93% (25). This fact shows that we are more aggressive in the young than in the elderly to achieve GTR explaining the higher rate of unfavorable rates in the Young STR/DS subcohort. In turn, the present analysis has shown that surgical management strategy in the elderly is more careful compared to the young.

Long-term results should be chosen to compare functional outcome between such different treatment modalities as radiosurgery and microsurgery, e.g., in hearing outcome. Watchful observation (“WaS”) abandons CN function (hearing and vestibular, more prominently then facial and trigeminal function) to the natural history of VS and tumor dynamic. Thus, it has been shown that 12% lose functional hearing in the course of the VS natural history (26). In line with this, SRS has been shown to result in a long-term hearing preservation of only 35%–51% (27) and therefore similarly to surgical treatment. Additionally, it is well known that hearing function dramatically decreases within the first decade after SRS (8, 13, 27). Additionally, tinnitus and imbalance are shown to increase and facial nerve dysfunction (e.g., hemispasm) might appear after SRS (28). Finally, little is known about radiation-associated tumor malignization (11, 29). The present study design, however, does not allow a direct comparison of functional outcome between radiotherapy and surgery.

It is not to be forgotten that functional outcome, as physicians and/or treating surgeons may define it, does not necessarily transfer to quality-of-life in the patients’ eyes in a proportional way. Leaving residual tumor behind or treating the tumor by non-invasive treatments such as SRS can impact mental health or illness perception (30).

### Extent of resection and recurrence-free survival

Due to the study design (matched by tumor size and the EOR), this analysis does not allow any general statement considering the frequency of the achieved EOR in the elderly compared to the young alone. However, when put into context with another series published and treated by the same group and institution with N = 572 primary VS patients, the rate of GTR was significantly higher among a general VS patient cohort of any age. Therefore, we can assume that the management of elderly patients with VS is carried out more conservatively—even in regard to the surgical EOR (25).

Mean time to recurrence was >5 years in both groups, proposing that long-term follow-up in VS patients should be carried out regardless of age. Still, the overall incidence of and mean time to recurrence were statistically insignificant in the elderly compared to the young concordant to previous matched cohort studies with distinctly smaller patient numbers (3, 10). In large VS, where surgery is inevitable, tumor mass reduction with SRS or observation could be suggested to avoid functional deterioration (9). In our cohort, microsurgical care followed by observation was the only treatment (no adjuvant SRS) carried out. Even though the number of STR- and DS-resected patients was low, the risk for tumor recurrence within 5 years was significantly increased in STR and DS. Thus, a deliberate DS as a standard treatment strategy in VS must be critically analyzed, especially in a setting of DS with observation alone (9). Therefore, if DS is inevitable, adjuvant therapy, e.g. SRS, should be evaluated for long-term tumor control. However, our study design does not allow any direct comparison between surgical GTR and DS plus SRS. This issue has yet to be investigated. Still, our results imply that if surgery is indicated in VS—even in patients with advanced age—GTR (or near-total tumor resection) should be the intention of surgery to ensure maximal tumor control. Also, our data imply that subtotal VS resection without adjuvant therapy is not recommended.

The EOR is significantly associated with RFS in both groups independent of age with noted early tumor recurrence in DS compared to STR and GTR. Pre-existing data show that the volume of residual tumor correlates with the incidence of recurrence (31, 32). GTR—whenever safely feasible—should be the primary intention-to-treat, and this study confirms the beneficial aspect in the tumor control of GTR compared to STR/DS even in an elderly cohort. We reckon the fact that STR is defined more strictly (minimal residual only in the IAC and complete removal of the tumor in the CPA) in this presented study than in previously published studies by other groups. Therefore, we suggest that the EOR must be defined homogenously to truly convey this observed relationship of the EOR, recurrence, and functional outcome to clinical day-to-day care in the form of intention-to-resect to patients’ benefit.

A definite statement on surgical treatment of VS is generally very difficult to acquire due to several reasons: (1) the level of evidence: there are no published randomized controlled clinical trials or even prospective studies on surgical resection in the current literature (11), (2) heterogenicity in surgical modality (e.g., approaches) by a heterogenous groups of specialists (neurosurgery and ENT), and 3) the lack of agreement of an EOR classification. To address these issues, an interdisciplinary network should be encouraged and a clinically relevant EOR classification should be enforced (including GTR, NTR, STR, and DS) to homogenously evaluate RFS and perioperative morbidity in larger multicenter settings. Such detailed distinction between the EOR might appear overelaborate, however, as tumor recurrence has shown to be dependent on the residual tumor, the idea of exact EOR classification has shown promise in other brain tumor entities already (33, 34).

### Limitations of this study

It is apparent that the retrospective nature of this study bears its limitations and biases. Firstly, this is a single-center study. Therefore, the generalizability and reproducibility of the results may be limited to specialized centers with a comparative caseload in VS. Furthermore, although the number of patients classified as elderly may be regarded as the largest cohort compared to previously published studies (3, 10), still, the patient number and its value have to be put into its statistical context. Moreover, detailed subgroup analysis cannot be carried out (e.g., tumor size and cystic morphology) due to this cohort size.

## Conclusion

The evidence level of VS management is remarkably low, and the debate on the risk–benefit ratio of surgical treatment is still ongoing, especially in the elderly. The present matched-cohort study shows that the microsurgical tumor resection of VS is safe and does not bear additional perioperative morbidity or worse functional outcome in the elderly as compared to a young control group. The overall incidence and of recurrence was statistically insignificant in the elderly compared to the young. When treating with surgery alone, the EOR determines RFS and the incidence of recurrence/progression in both study cohorts. For maximal tumor control, GTR should be intended. As incidence of recurrence is not significantly lower compared to the young. Postoperative follow-up should be carried out as mean time to recurrence was > 5 years in both groups. If leaving relevant tumor residual is inevitable, adjuvant therapy, e.g. SRS, should be evaluated in the Elderly.

## Data availability statement

The raw data supporting the conclusions of this article will be made available by the authors, without undue reservation.

## Ethics statement

Ethical review and approval were not required for the study on human participants in accordance with the local legislation and institutional requirements. Written informed consent from the participants was not required to participate in this study in accordance with the national legislation and the institutional requirements.

## Author contributions

SW contributed to the acquisition, analysis, interpretation of data, conception and design, and writing the first draft. KM and FE contributed to interpretation of the data and critical review of the final manuscript. GN contributed to data analysis, interpretation of the data, and reviewing the final manuscript. MT contributed to the interpretation of the data and critical review of the manuscript. All authors contributed to the article and approved the submitted version.
